# The impact of gender and the social determinants of health on the clinical course of people living with HIV in Myanmar: an observational study

**DOI:** 10.1186/s12981-021-00364-w

**Published:** 2021-08-09

**Authors:** Phyo Pyae Nyein, Eithandee Aung, Ne Myo Aung, Mar Mar Kyi, Mark Boyd, Kyaw Swar Lin, Josh Hanson

**Affiliations:** 1 Mingaladon Specialist Hospital, Mingaladon Township, Yangon, Myanmar; 2 University of Medicine 2, North Okkalapa Township, Yangon, Myanmar; 3 The Kirby Institute, University of New South Wales Sydney, Sydney, Australia; 4 Insein General Hospital, Insein Township, Yangon, Myanmar; 5 Faculty of Health and Medical Sciences, University of Adelaide, Adelaide, Australia

**Keywords:** HIV, Acquired immunodeficiency syndrome, Social determinants of health, Myanmar, Gender

## Abstract

**Background:**

There is a growing recognition of the impact of gender and the social determinants of health on the clinical course of people living with HIV (PLHIV). However, the relative contribution of these factors to clinical outcomes of PLHIV is incompletely defined in many countries. This study was performed to gain a greater understanding of the non-clinical determinants of prognosis of PLHIV in Myanmar.

**Methods:**

Selected demographic, behavioural and socioeconomic characteristics of outpatients at two specialist HIV hospitals and one general hospital in Yangon, Myanmar were correlated with their subsequent clinical course; a poor outcome was defined as death, hospitalisation, loss to follow-up or a detectable viral load at 6 months of follow-up.

**Results:**

221 consecutive individuals with advanced HIV commencing anti-retroviral therapy (ART) were enrolled in the study; their median CD4 T-cell count was 92 (44–158) cells/mm^3^, 138 (62.4%) were male. Socioeconomic disadvantage was common: the median (interquartile range (IQR) monthly per-capita income in the cohort was US$48 (31–77); 153 (69.9%) had not completed high school. However, in a multivariate analysis that considered demographic, behavioural, clinical factors and social determinants of health, male gender was the only predictor of a poor outcome: odds ratio (95% confidence interval): 2.33 (1.26–4.32, p = 0.007). All eight of the deaths and hospitalisations in the cohort occurred in males (p = 0.03).

**Conclusions:**

Men starting ART in Myanmar have a poorer prognosis than women. Expanded implementation of gender-specific management strategies is likely to be necessary to improve outcomes.

**Supplementary Information:**

The online version contains supplementary material available at 10.1186/s12981-021-00364-w.

## Introduction

In 2019, the HIV prevalence in individuals aged 15–49 in Myanmar was estimated to be 0.7%, one of the highest in Southeast Asia [1]. However, over the past decade there has been significant progress in the care of people living with HIV (PLHIV) in Myanmar. Antiretroviral therapy (ART) coverage is estimated to have risen from 17% in 2011 to 76% in 2019, while over the same time period the annual number of AIDS-related deaths nationally is estimated to have fallen from 11,000 to 7700 [1]. Once on ART, most patients do well: in one cohort—where the median CD4 count was only 169 cells/mm^3^—the 12-month mortality was as low as 5.7% [2]. However, these encouraging outcomes are not seen uniformly: other studies have reported attrition rates (a combination of death within 6 months and loss to follow-up (LTFU)) of up to 20%) [3]. This attrition was associated with a variety of factors including gender, marital status, level of education and hazardous alcohol consumption [3].

There is a growing recognition of the relationship between the social determinants of health (SDH) and clinical outcomes in PLHIV [4]. This is particularly the case in countries like Myanmar, where, in 2017, a quarter of the population lived in poverty, where one in ten adults has never attended school, and where for another 53%, primary education–either completed or uncompleted – is the highest level of educational attainment [5].

Gender also has a significant impact on the clinical outcomes of PLHIV [6]. Globally, prevailing concepts of masculinity lead to men being less likely than women to seek out health care, less likely to have HIV testing and less likely to initiate and adhere to ART [7]. All of these factors conspire to result in significantly higher mortality in men [8]. Similar concepts of masculinity exist in Myanmar and have an important impact on the way in which men access health care [9, 10]. While gender has been linked to poorer outcomes in men living with HIV in Myanmar [3], its independent contribution to clinical outcomes is incompletely defined.

This pilot study was performed to determine the demographic and socioeconomic characteristics of PLHIV in Myanmar and the contribution of these factors to short-term morbidity and mortality. The study’s objective was to gain a greater understanding of the non-clinical determinants of prognosis to inform a more comprehensive approach to the local care of PLHIV.

## Methods

This study included participants of a randomized controlled trial (RCT) which was being performed at 3 ART clinics (Specialist Hospital Mingaladon, Specialist Hospital Waibargi and Insein General Hospital) in Yangon, Myanmar’s largest city.

The RCT examines immediate versus deferred isoniazid preventive therapy (IPT) in PLHIV with advanced disease. The RCT commenced in January 2018 and is continuing at the time of writing. Participants were eligible for the RCT if they were ART naïve adults (≥ 18 years) with advanced HIV (CD4 cell count < 200 cells/mm^3^ or WHO stage 3 or 4 disease), with no contraindication to IPT (symptoms of active TB, abnormal liver function tests (serum bilirubin > 20 µmol/L or alanine transferase > 45 IU/mL), peripheral neuropathy or allergy to isoniazid).

RCT participants were identified in the outpatient ART clinics of the three study sites and enrolled into the study after written, informed consent. Between January and October 2019, participants were also invited to participate in this study. After study doctors obtained written, informed consent for this substudy, demographic, socioeconomic, and selected clinical data were collected using a dedicated *pro forma*. Household income data were collected in Myanmar Kyat (MMK), but are expressed here in United States Dollars (USD) using an exchange rate of 1 USD to 1300MMK. The participants were then followed sequentially. The primary endpoint for the study was a poor clinical outcome, which was defined as death, hospitalisation, loss to follow-up or a detectable viral load (≥ 40 copies/mL) at 6 months.

Data were de-identified, entered into an electronic database (Additional file 1) and analysed using statistical software (Stata version 14.2). Univariate analysis was performed using the Kruskal–Wallis, chi-squared or Fisher’s exact test where appropriate. Multivariate analysis was performed using backwards stepwise regression using variables that were significant with a p < 0.30 in univariate analysis. If individuals were missing data, they were not included in analyses which evaluated those variables.

The University of Medicine 2 Yangon Ethics Review Committee provided ethical approval for the study (approval number 105/ERC-3 [5/2019]).

## Results

There were 221 patients enrolled into this study, their median (interquartile range, IQR) age was 37 (32–46) and 138 (62.4%) were male. Their baseline characteristics and clinical course, stratified by gender, are presented in Table 1.

**Table 1:** Differences in the baseline characteristics and clinical course of male and female participants

Variable	Male n = 138	Female n = 83	p
Age	37 (31–46)	36 (33–44)	0.95
Unmarried	66 (48%)	40 (48%)	0.96
Unemployed	19 (14%)	34 (41%)	< 0.001
Living outside Yangon	38 (28%)	16 (19%)	0.17
Did not complete high school	93 (68%)	60 (72%)	0.54
Number of family members living at home	3 (2–4)	3 (2–5)	0.90
Per-capita household income (USD)	46.15 (30.76–76.92)	51.28 (28.04–76.92)	0.90
Hazardous alcohol consumption	47/137 (34%)	0/83	< 0.0001
Current cigarette smoker	54/128 (42%)	2/83 (2%)	< 0.0001
Active injecting drug use	3/138 (2%)	0/83	0.29
Presently chewing betel nut	70/118 (59%)	7/82 (9%)	< 0.0001
Hepatitis B surface antigen positive	15/127 (12%)	6/79 (8%)	0.48
Hepatitis C antibody positive	4/127 (3%)	2/79 (3%)	1.0
Body mass index (kg/m^2^)	19.3 (17.2–22.2)	18.9 (16.1–22.3)	0.29
Mean arm circumference (cm)	25 (22–27)	25 (22–27)	0.99
WHO stage at enrolment	1 (1–3)	2 (1–3)	0.02
CD4 cell count (cells/mm^3^)	92 (41–157)	92 (48–158)	0.61
Receiving dolutegravir containing regimen	117/137 (85%)	64/82 (78%)	0.16
Haemoglobin (g/dL)	12 (10–13.2)	10 (9–11)	0.0001
Creatinine (µmol/L)	95 (80–113)	80 (70–100)	0.0004
Died^a^	6/138 (4%)	0/83	0.09
Hospitalised^a^	2/138 (1%)	0/83	0.53
Died or hospitalised^a^	8/138 (6%)	0/83	0.03
Lost to follow-up^a^	10/138 (7%)	6/83 (7%)	1.0
Detectable virus^a^	53/99 (54%)	20/61 (33%)	0.01

All numbers represent absolute numbers (%) or median (interquartile range)

^a^At 6 months’ follow-up

The demographic and socioeconomic characteristics of the men and women were similar. The median (IQR) age of the men was 37 (31–46) compared with 36 (33–44) among women (p = 0.95). A similar proportion of men and women were unmarried (66/138 (47.8%) versus 40/83 (48.2%), p = 0.95), a similar proportion had completed high school (43/136 (31.6%) versus 23/83 (27.7%), p = 0.54) and a similar proportion lived outside Yangon (38/138 (27.5%) versus 16/83 (19.3%), p = 0.17). The number of people living at home was comparable for men and women (median (IQR): 3 (2–4) versus 3 (2–5), p = 0.90) as was the monthly per-capita income (median (IQR): USD 46.15 (30.77–76.92) versus USD 51.28 (28.04–76.92), p = 0.90).

However, men were more likely to be employed than women (119/138 (86.2%) versus 49/83 (59.0%), p < 0.0001). Men were also more likely to report hazardous alcohol use (47/137 (34.1%) versus 0/83, p < 0.0001), more likely to smoke tobacco (54/128 (42.2%) versus 2/83 (2.4%), p < 0.0001) and to chew betel (70/118 (59.3%) versus 7/82 (8.5%), p < 0.0001). The difference in intravenous drug use (3/138 (2.2%) versus 0/83) did not reach statistical significance (p = 0.29).

The median (IQR) Body Mass Index (BMI) of men and women was similar (19.3 (17.2–22.2) versus 18.9 (16.1–22.3) kg/m^2^, p = 0.29) as was the mid-upper arm circumference (MUAC) (25 (22–27) versus 25 (22–27) cm, p = 0.99). The median CD4 cell count was comparable (92 (41–157) versus 92 (48–158) cells/mm^3^, p = 0.61), however men had less advanced clinical disease at enrolment (median (IQR) WHO stage 1 (1–3) versus 2 (1–3), p = 0.02) and a higher haemoglobin level (median (IQR) 12 (10–13.2) versus 10 (9–11) g/dL, p = 0.0001).

Of the 221 patients enrolled in the study, there were 40 (18.1%, 24 male and 16 female) patients who were not able to have 6-month viral load testing; in 13 (32.5%) this was due to the evolving global COVID-19 pandemic which prevented clinic attendance for testing. Although none of these 40 patients died, were hospitalised, or were lost to follow-up, the absence of follow-up virological assessment precluded analysis of these patients using the pre-specified primary endpoint.

**Fig. 1:**
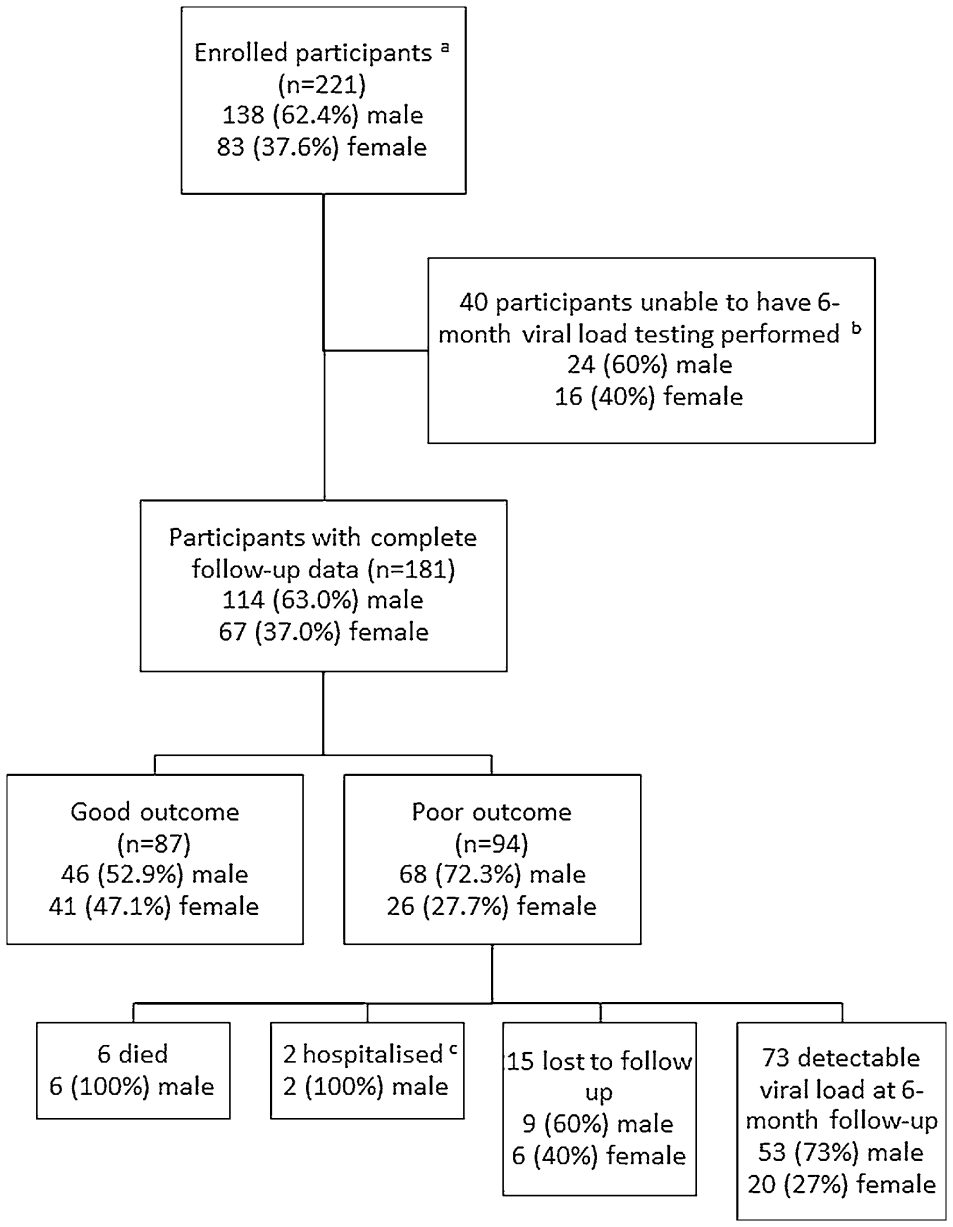
Clinical course of the participants. ^a^ART naïve participants with HIV eligible for IPT. ^b^All 40 participants were alive, remained retained in care and had no hospitalisations after 6 months of follow-up. ^c^Of the 2 hospitalised patients, one was also subsequently lost to follow-up and one also had a detectable viral load at 6 months

Among the remaining 181 patients, 94 (52%) had a poor outcome (Fig. 1). This was death in 6/181 (3.3%), hospitalisation without death in 2/181 (1.1%), loss to follow-up in 16/181 (8.8%) and a detectable viral load in 73/181 (40.3%). There were 3/181 (1.7%) who had month 6 viral load of > 1000 copies/ml.

All the deaths and hospitalisations occurred among men (8/138 (5.5%) versus 0/83 women, p = 0.03), but there was no gender difference in loss to follow-up (10/138 (7.3%) versus 6/83 (7.2%), p = 1.0). A detectable viral load was also more common in men than women (55/90 (53.5%) versus 20/61 (23.8%), p = 0.01). Gender was the only predictor of a poor outcome in univariate analysis (Table 2). Even in multivariate analysis that also considered the contribution of education level, income, employment, residence in Yangon, hazardous alcohol consumption, CD4 cell count and ART regimen, only gender predicted a poor outcome (odds ratio (95% confidence interval): 2.3 (1.26–4.32), p = 0.007).

**Table 2:** Baseline socio-demographic and clinical characteristics of participants and 6-month outcome

Variable	All patients n = 221	Poor outcome^a^ n = 94	Good outcome n = 87	p
Age	37 (32–46)	39 (33–47)	36 (33–44)	0.29
Male gender	138 (62%)	68 (72%)	46 (53%)	0.007
Unmarried	106 (48%)	45 (48%)	45 (52%)	0.61
Unemployed	53 (24%)	21 (22%)	20 (23%)	0.92
Living outside Yangon	54 (24%)	25 (27%)	18 (21%)	0.35
Did not complete high school	153 (70%)	64/92 (70%)	62/87 (71%)	0.80
Number of family members living at home	3 (2–4)	3 (2–4)	4 (2–5)	0.49
Per-capita household income (USD)	48.07 (30.77–76.92)	46.15 (30.77–76.92)	51.28 (28.04–76.92)	0.31
Hazardous alcohol consumption	47/220 (21%)	20/94 (21%)	15/86 (17%)	0.52
Current cigarette smoker	56/211 (27%)	27/89 (30%)	16/82 (20%)	0.12
Active injecting drug use	3 (1%)	1 (1%)	2 (2%)	0.61
Presently chewing betel nut	77/200 (39%)	37/84 (44%)	26/77 (34%)	0.18
Hepatitis B surface antigen positive	21/206 (10%)	7/89 (8%)	7/77 (9%)	0.79
Hepatitis C antibody positive	6/206 (3%)	2/89 (2%)	2/77 (3%)	1.0
Body mass index (kg/m^2^)	19.1 (17.0–22.3)	19.5 (16.6–22.6)	19.5 (17.3–22.1)	0.77
Mean arm circumference (cm)	25 (22–27)	25 (22–28)	24 (22–27)	0.70
WHO stage at enrolment	1 (1–3)	1 (1–3)	1 (1–2)	0.25
CD4 cell count (cells/mm^3^)	92 (44–158)	86 (41–145)	99 (47–158)	0.25
Receiving dolutegravir containing regimen	181/219 (83%)	78/93 (84%)	76/87 (87%)	0.51
Haemoglobin (g/dL)	11.0 (9.6–12.8)	10.4 (9.6–12.2)	11.0 (9.7–12.9)	0.33
Creatinine (µmol/L)	88 (75–107)	91 (77–111)	91 (75–107)	0.61

All numbers represent absolute numbers (%) or median (interquartile range)

^a^Defined as death, hospitalisation, loss to follow-up or detectable viral load at 6 months

## Discussion

This small study highlights the significant impact of gender on the clinical course of PLHIV in Yangon, Myanmar. Although men had less advanced disease at enrolment, all the deaths and hospitalisations that were seen in the cohort during follow-up occurred in men. A detectable viral load at 6-month follow-up was also more common in men.

The cohort represented a significantly disadvantaged population: less than one-third had completed high school, almost one-quarter were unemployed, and the median (IQR) per-capita monthly income was only USD 48 (31–77)) in a country where the median per-capita monthly income is USD 51 [5]. However, it was notable that there was no association between any of these indices and the pre-defined clinical endpoints in either univariate or multivariate analysis.

In contrast, the association between gender and outcomes seen in the cohort was striking. The association between male gender and poor outcome in PLHIV is consistent with previous work performed in Myanmar and other countries [3, 6, 11–13]. Men in sub-Saharan Africa living with HIV have been found to have poorer engagement than women at every step of the treatment cascade. They have lower rates of testing, later ART initiation, inferior ART adherence and poorer retention in care, all of which results in significantly worse HIV-related outcomes [6, 13, 14].

This is a pattern seen in numerous health conditions, not just HIV. Despite the fact that in many countries men generally have significantly greater privilege and opportunity than women, this advantage does not necessarily translate into better health outcomes [15]. The most commonly cited explanation is the impact of behaviours associated with conventional masculine gender norms and practices. For many men, the concept of masculinity not only encourages activities that increase the risk of disease, but makes reporting of symptoms and seeking healthcare less likely [15]. Indeed, the more closely men identify with conventional notions of masculinity, the more likely they are to exhibit damaging lifestyle behaviours and avoid services [16]. While the participants’ concepts of masculinity were not explored in this study, there was a striking gender imbalance in hazardous alcohol consumption, cigarette smoking and betel chewing. It is also important to emphasise that in Myanmar society, there are pervasive expectations of feminine behaviour, with drug and alcohol use particularly discouraged [10]. However, it should be noted that while all the injecting drug use and hazardous alcohol consumption—and greater than 95% of the cigarette smoking—was reported by men in the cohort, none of these factors were independently associated with the primary endpoint in multivariate analysis.

It is estimated that 87% (78–95) of women ≥ 15 are receiving ART in Myanmar compared with 70% (62–80) of men ≥ 15 years of age [1]. Health-seeking behaviour was not evaluated in this cohort, but some of the patterns seen in African countries are likely to be present in Myanmar and these are likely to be exacerbated by social and political barriers. Young women in Myanmar may be more likely to access the health system–and routinely screened for HIV–during antenatal and obstetric care. Meanwhile men who have sex with men (MSM) may be less inclined to seek healthcare due to concerns about discrimination and stigmatization in a country where same-sex intercourse remains illegal [17]. In a country where between 4 and 32% of MSM have disclosed to their family that they have sex with men, it is estimated that only 44% of MSM are receiving ART [18]. In Myanmar, men are more likely to travel to other regions for work and therefore might be expected to have a higher loss to follow-up rate although there was no significant gender difference in loss-to-follow-up in this cohort.

Even though the study only enrolled patients with advanced disease—who were generally profoundly socioeconomically disadvantaged—six months after starting ART, less than 3% had died and less than 7% lost to follow up. This suggests that once patients in Myanmar are able to access ART they usually do well. This may be explained by the fact that all HIV care in Myanmar is free of charge, that there is increasing access to contemporary ART (over 80% of this cohort received a dolutegravir-based regimen) and that medication adherence is generally excellent [3, 19]. The Global Fund to fight AIDS, Tuberculosis and Malaria provides funding for peer support workers who support HIV care, provide counselling and psychosocial support and who play a crucial role in ensuring retention in care [3]. Recent implementation of an open-source electronic Medical Record System has improved data collection and has the potential to further facilitate care [3]. However, the fact that a gender-gap in outcomes persists despite these interventions suggests that strategies tailored to the specific needs of the male PLHIV in Myanmar are necessary to further improve outcomes. Greater access to self-testing [20], the creation of specific clinics that recognise the differences in the way that men access healthcare [21], and even financial incentives [22, 23] have been used successfully in other parts of world to improve engagement with care. However, the feasibility and sustainability of these interventions in Myanmar needs to be established and would be the basis for future research [8, 17].

This study has limitations. The lack of association between the social determinants of health and outcomes seen in this small cohort (which increases the likelihood of a type 2 error) does not suggest that these factors can be ignored. There is a significant body of work that demonstrates their importance and larger studies have shown their impact on HIV-related outcomes [3, 11, 24]. It is likely that a limited range of values for at least some of the variables–the standard deviation in monthly per-capita income was only USD68–also contributed to this lack of statistical significance. The exclusion of patients with active tuberculosis—or symptoms suggestive of active tuberculosis—would also be expected to affect the number of deaths and hospitalisations during follow up [25]. A failure to enquire about MSM behaviour precluded analysis of the contribution that any resulting stigmatization and discrimination may have made to the clinical outcomes of the male participants [17]. However, in a country where homosexuality remains criminal, there were concerns about the impact that addressing this subject might have on the participants’ confidence in receiving ongoing care at the study hospital [26]. It could be argued that a detectable viral load is too stringent an endpoint and that the World Health Organization cut-off of 1000 copies/mL might be preferable, although there are recent data to suggest that this level is too high to identify patients at risk of virological failure [27, 28]. However, if viral load was excluded from the analysis and only the “harder” endpoints of death of hospitalisation were included, gender–but none of the SDH–was still associated with outcome. Finally, while the contribution of gender and the SDH to outcomes was determined using multivariate analysis, some of the variables’ definitions—for instance, the binary “employed or unemployed”—were crude. More detailed description of these variables and their relationship with health-related behaviours might be expected to result in a more nuanced analysis of their impact on outcomes [29]. However, at the very least the study provides hypothesis-generating data that might be explored in future studies.

## Conclusions

Socioeconomic disadvantage was common in this cohort of PLHIV in urban Myanmar, however conventional SDH were not associated with important clinical outcomes during 6-months of follow-up. Gender had more prognostic utility and is likely linked to health-related behaviours that result from community level gender norms in Myanmar. Addressing and modifying these gender norms is challenging, however these data demonstrate that health care workers in Myanmar may need to provide HIV care that recognizes the contribution of gender to the clinical course if they are to deliver optimal outcomes.

## Supplementary Information

**Additional file 1.** Dataset.

## Data Availability

The dataset supporting the conclusions of this article is included within the article (and its additional files).

## Abbreviations

HIV: Human Immunodeficiency Virus
ART: Anti-retroviral therapy
PLHIV: People living with HIV
LTFU: Lost to follow up
RCT: Randomised controlled trial
IPT: Isoniazid prophylaxis therapy
WHO: World Health Organization
MMK: Myanmar Kyats
USD: United States Dollars
IQR: Interquartile range
BMI: Body mass index

## References

[1] UNAIDS Country Fact Sheets: Myanmar: UNAIDS; 2019 https://www.unaids.org/en/regionscountries/countries/myanmar.

[2] Aung NM, Hanson J, Kyi TT, Htet ZW, Cooper DA, Boyd MA (2017). HIV care in Yangon, Myanmar; successes, challenges and implications for policy. AIDS Res Ther.

[3] Aung ZZ, Oo MM, Tripathy JP, Kyaw NTT, Hone S, Oo HN (2018). Are death and loss to follow-up still high in people living with HIV on ART after national scale-up and earlier treatment initiation? A large cohort study in government hospital-based setting, Myanmar: 2013–2016. PLoS ONE.

[4] Closing the gap in a generation: health equity through action on the social determinants of health. Final report of the Commission on Social Determinants of Health. Geneva; 2008. 10.1016/S0140-6736(08)61690-6 18994664

[5] Myanmar Living Conditions Survey 2017: Socio-economic Report. Nay Pyi Taw and Yangon, Myanmar; 2020.

[6] Cornell M, Johnson LF, Wood R, Tanser F, Fox MP, Prozesky H (2017). Twelve-year mortality in adults initiating antiretroviral therapy in South Africa. J Int AIDS Soc.

[7] Blind spot. Reaching out to men and boys. Addressing a blind spot in the response to HIV. Geneva; 2017.

[8] Colvin CJ (2019). Strategies for engaging men in HIV services. Lancet HIV.

[9] Lakin DP, Win KS, Aung H, Soe KNC, Kyi B, Marcell AV (2021). Masculinity and mental health treatment initiation for former political prisoners in Yangon, Myanmar-a qualitative investigation. BMC Public Health.

[10] Htet H, Saw YM, Saw TN, Htun NMM, Lay Mon K, Cho SM (2020). Prevalence of alcohol consumption and its risk factors among university students: A cross-sectional study across six universities in Myanmar. PLoS ONE.

[11] Dalhatu I, Onotu D, Odafe S, Abiri O, Debem H, Agolory S (2016). Outcomes of Nigeria's HIV/AIDS treatment program for patients initiated on antiretroviral treatment between 2004–2012. PLoS ONE.

[12] Telele NF, Kalu AW, Marrone G, Gebre-Selassie S, Fekade D, Tegbaru B (2018). Baseline predictors of antiretroviral treatment failure and lost to follow up in a multicenter countrywide HIV-1 cohort study in Ethiopia. PLoS ONE.

[13] Tsai AC, Siedner MJ (2015). The Missing Men: HIV Treatment Scale-Up and Life Expectancy in Sub-Saharan Africa. PLoS Med.

[14] Kerkhoff AD, Sikombe K, Eshun-Wilson I, Sikazwe I, Glidden DV, Pry JM (2020). Mortality estimates by age and sex among persons living with HIV after ART initiation in Zambia using electronic medical records supplemented with tracing a sample of lost patients: A cohort study. PLoS Med.

[15] Baker P, Dworkin SL, Tong S, Banks I, Shand T, Yamey G (2014). The men's health gap: men must be included in the global health equity agenda. Bull World Health Organ.

[16] Baker P (2019). Improving men's health: successful initiatives and barriers to progress. Br J Nurs.

[17] Veronese V, Traeger M, Oo ZM, Tun TT, Oo NN, Maung H (2020). HIV incidence and factors associated with testing positive for HIV among men who have sex with men and transgender women in Myanmar: data from community-based HIV testing services. J Int AIDS Soc.

[18] Myanmar Integrated Biological and Behavioural Surveillance Survey. National AIDS Program, Ministry of Health and Sports, Myanmar. 2019.

[19] Myint N, Zaw TT, Sain K, Waiyan S, Danta M, Cooper D (2020). Sequential Helicobacter pylori eradication therapy in Myanmar; a randomized clinical trial of efficacy and tolerability. J Gastroenterol Hepatol.

[20] Guy RJ, Prestage GP, Grulich A, Holt M, Conway DP, Jamil MS (2015). Potential public health benefits of HIV testing occurring at home in Australia. Med J Aust.

[21] Addressing the Blind Spot in Achieving Epidemic Control in Malawi: Implementing "male-friendly" HIV services to increase access and uptake: PEPFAR Solutions Platform; 2018 https://www.pepfarsolutions.org/solutions/2018/12/19/addressing-the-blind-spot-in-achieving-epidemic-control-in-malawi-implementing-male-friendly-hiv-services-to-increase-access-and-uptake.

[22] Kennedy CE, Yeh PT, Atkins K, Fonner VA, Sweat MD, O'Reilly KR (2020). Economic compensation interventions to increase uptake of voluntary medical male circumcision for HIV prevention: A systematic review and meta-analysis. PLoS ONE.

[23] Nglazi MD, van Schaik N, Kranzer K, Lawn SD, Wood R, Bekker LG (2012). An incentivized HIV counseling and testing program targeting hard-to-reach unemployed men in Cape Town, South Africa. J Acquir Immune Defic Syndr.

[24] Socio-economic I, Euro-Coord HIVWGfCoOHERiEi. Inequalities by educational level in response to combination antiretroviral treatment and survival in HIV-positive men and women in Europe. AIDS. 2017;31(2):253–62. 10.1097/QAD.0000000000001270 27662557

[25] Thit SS, Aung NM, Htet ZW, Boyd MA, Saw HA, Anstey NM (2017). The clinical utility of the urine-based lateral flow lipoarabinomannan assay in HIV-infected adults in Myanmar: an observational study. BMC Med.

[26] Veronese V, Clouse E, Wirtz AL, Thu KH, Naing S, Baral SD (2019). "We are not gays don't tell me those things": engaging 'hidden' men who have sex with men and transgender women in HIV prevention in Myanmar. BMC Public Health.

[27] Amstutz A, Nsakala BL, Vanobberghen F, Muhairwe J, Glass TR, Namane T (2020). Switch to second-line versus continued first-line antiretroviral therapy for patients with low-level HIV-1 viremia: An open-label randomized controlled trial in Lesotho. PLoS Med.

[28] Consolidated guidelines on the use of antiretroviral drugs for treating and preventing HIV infection; Recommendations for a public health approach. Geneva; 2016. 27466667

[29] Cottrell EK, Hendricks M, Dambrun K, Cowburn S, Pantell M, Gold R (2020). Comparison of Community-Level and Patient-Level Social Risk Data in a Network of Community Health Centers. JAMA Netw Open..

